# Classification of acetic acid bacteria and their acid resistant mechanism

**DOI:** 10.1186/s13568-021-01189-6

**Published:** 2021-02-17

**Authors:** Xiaoman Qiu, Yao Zhang, Housheng Hong

**Affiliations:** 1 College of Biotechnology and Pharmaceutical Engineering, Nanjing Tech University, No. 30, Puzhu Road, Nanjing, 211800 China; 2 National Engineering Technique Research Center for Biotechnology, Nanjing Tech University, No. 30, Puzhu Road, Nanjing, 211800 China

**Keywords:** Acetic acid bacteria, Genus and species classification, Metabolic regulatory, Acid resistance mechanism, Quorum sensing, Signaling pathways

## Abstract

Acetic acid bacteria (AAB) are obligate aerobic Gram-negative bacteria that are commonly used in vinegar fermentation because of their strong capacity for ethanol oxidation and acetic acid synthesis as well as their acid resistance. However, low biomass and low production rate due to acid stress are still major challenges that must be overcome in industrial processes. Although acid resistance in AAB is important to the production of high acidity vinegar, the acid resistance mechanisms of AAB have yet to be fully elucidated. In this study, we discuss the classification of AAB species and their metabolic processes and review potential acid resistance factors and acid resistance mechanisms in various strains. In addition, we analyze the quorum sensing systems of *Komagataeibacter* and *Gluconacetobacter* to provide new ideas for investigation of acid resistance mechanisms in AAB in the form of signaling pathways. The results presented herein will serve as an important reference for selective breeding of high acid resistance AAB and optimization of acetic acid fermentation processes.

## Key points

- Summarize the current classification of AAB (19 genera and 92 species) in detail for the first time;
- Investigate the acid resistance mechanism in AAB systematically and comprehensively;
- Explain the acid resistance mechanism from the new perspective of signal pathways.

## Introduction

Acetic acid bacteria (AAB), which are also known as *Acetobacter* sp., are obligate aerobic Gram-negative bacteria found in the *Alphaproteobacteria* class, *Rhodospirillales* order, and *Acetobacteraceae* family (Kersters 2006). AAB are often found in warm and humid regions, in fruits, flowers, fruit fly guts, and some fermented foods (Chouaia et al. 2014; Kersters et al. 2006; Sengun and Karabiyikli 2011; Soemphol et al. 2011; Trček and Barja 2015). When compared with other bacteria, AAB show high variability (Azuma et al. 2009). Therefore, the taxonomy of AAB has undergone a long process of development that started with an initial phenotypic classification and continued as the polyphasic classification approach became available. Polyphasic classification mainly includes phenotypic, chemical, and genetic classification methods (Greenberg et al. 2006; Lisdiyanti et al. 2006). In the past few decades, the development of molecular biology techniques has further refined the biological classification of AAB. However, as things stand at present, no researchers have summarized the newly discovered specific genus and species classification of AAB systematically, except for a 2008 article that only summarized the 10 genus and 45 species (Cleenwerck and Vos 2008), which is a major focus of our article.

Major metabolic pathways in AAB include the ethanol oxidation respiratory chain pathway, tricarboxylic acid cycle pathway, pyruvate metabolic pathway, and pentose phosphate pathway. Among these, the most significant reaction is the incomplete oxidation of sugars, alcohols, or sugar alcohols into aldehydes, ketones, and organic acids (Sengun 2017). The greatest strength of AAB is their ability to use less biomass to produce large amounts of acetic acid compared to other bacterias that produce organic acids (López-Garzón and Straathof 2014); therefore, they are important industrial microorganisms that are widely used in the production of vinegar and fruit vinegar, gluconic acid products, and development of bio-fuel cells (Lynch et al. 2019; Misra et al. 2012; Sainz et al. 2016).

The presence of acetic acid in vinegar products makes AAB fermentation unique (Zhang et al. 2016; Zheng et al. 2018). Specifically, acetic acid changes the flavor of vinegar and increases the survival advantages of AAB (Lynch et al. 2019; Hong 2016, 2017); however, acetic acid accumulation causes acid stress that inhibits AAB growth (Trček et al. 2015). During fermentation, the large number of dehydrogenases on the cell membrane of AAB causes the incomplete oxidation of many carbon sources into acetic acid (Matsushita et al. 2016). Because of the incomplete glycolysis, the main energy sources for maintaining cellular homeostasis are from the respiratory chain, tricarboxylic acid cycle, and pentose phosphate pathway (Illeghems et al. 2013). Resistance towards highly acidic environments requires large amounts of energy, which severely limits cell growth. As a result, AAB with high acid resistance can increase acetic acid productivity and conversion rate, thereby increasing the bioconversion efficiency of acetic acid. Hence, elucidation of acid resistance mechanisms can provide important guidance for the selective breeding of acetic acid-producing bacteria and bioconversion of high acidity vinegar.

We found that most researchers only wrote part of the acid resistance mechanism of AAB and none of them described the relationship between the quorum sensing and acid resistance mechanism of AAB. In this review, we discuss the specific classification of AAB for the first time and its metabolic pathways before systematically and comprehensively summarizing the latest studies on acid resistance in AAB. In addition, we analyze the quorum sensing systems of *Komagataeibacter* and *Gluconacetobacter* to elucidate acid resistance mechanisms in AAB from a new perspective of signal pathways.

### Overview of AAB and its taxonomy

There are many types of AAB, among which the first genus, *Acetobacter*, was first proposed and described by Beijerinck in 1898 (Beijerinck 1898). Subsequently, four major genera (*Acetobacter*, *Gluconobaeter*, *Gluconacetobacter*, and *Komagataeibacter*) were confirmed based on their ethanol oxidation capabilities and the type of respiratory chain coenzyme they contained (Asai 1935; Yamada et al. 1983, 2012). With the development of polyphasic classification techniques, new genera and species have been continuously found (Cleenwerck 2008), and 19 genera and 92 species of AAB have been identified to date (Table 1). AAB are mainly used in the industrial production of vinegars and fruit vinegar beverages, with *Acetobacter* and *Komagataeibacter* being primarily used in vinegar making (Kanchanarach et al. 2010; Wu et al. 2012).

**Table 1:** Current classification of the *Acetobacteraceae* (19 genera, 92 species)

Species^a^	DNA G + C(mol%)^b^	References	Species^a^	DNA G + C(mol%)^b^	References
***Acetobacter aceti***	56.4–58.3	Lisdiyanti et al. (2000)	*Gluconacetobacter diazotrophicus*	61.0–63.0	Yamada et al. (1997)
*Acetobacter ascendens*	53.2–53.3	Kim et al. (2018)	*Gluconacetobacter entanii*	58.0	Lisdiyanti et al. (2006)
*Acetobacter cerevisiae*	56.0–57.6	Iino et al. (2012)	*Gluconacetobacter johannae*	57.96–67.5	Nishijima et al. (2013)
*Acetobacter cibinongensisc*	53.8–54.5	Lisdiyanti et al. (2001)	*Gluconacetobacter liquefaciens*	63.5–66.9	Yamada et al. (1997)
*Acetobacter estunensis*	59.2–60.2	Lisdiyanti et al. (2000)	*Gluconacetobacter sacchari*	62.1–67.3	Franke et al. (1999)
*Acetobacter fabarum*	56.8–58	Iino et al. (2012)	*Gluconacetobacter takamatsuzukensis*	66.6	Nishijima et al. (2013)
*Acetobacter farinalis*	56.3–56.5	Iino et al. (2012)	*Gluconacetobacter tumulicola*	64.7	Nishijima et al. (2013)
*Acetobacter ghanensis*	56.9–57.3	Iino et al. (2012)	*Gluconacetobacter tumulisoli*	66.5	Nishijima et al. (2013)
*Acetobacter indonesiensis*	53.7–55.0	Lisdiyanti et al. (2000)	*Gluconobacter albidusf*	58.1–60.0	Malimas et al. (2007)
*Acetobacter lambici*	56.2	Spitaels et al. (2013)	*Gluconobacter cerinus*	54–56	Malimas et al. (2007)
*Acetobacter lovaniensis*	57.1–58.9	Iino et al. (2012)	*Gluconobacter frateurii*	57.5–57.7	Malimas et al. (2007)
*Acetobacter malorum*	57.2	Iino et al. (2012)	*Gluconobacter japonicus*	56.4	Malimas et al. (2009)
*Acetobacter musti*	58	Ferrer et al. (2016)	*Gluconobacter kanchanaburiensis*	59.5	Tanasupawat et al. (2011)
*Acetobacter nitrogenifigens*	64.1	Dutta and Gachhui (2006)	*Gluconobacter kondonii*	59.8	Malimas et al. (2007)
*Acetobacter oeni*	58.1	Silva et al. (2006)	*Gluconobacter nephelii*	57.2–57.6	Kommanee et al. (2010)
*Acetobacter okinawensis*	59.2–59.4	Iino et al. (2012)	*Gluconobacter oxydans*	60.3–63.5	Malimas et al. (2007)
*Acetobacter orientalis*	52.0–52.8	Lisdiyanti et al. (2001)	*Gluconobacter roseus*	60.5	Tanasupawat et al. (2011)
*Acetobacter orleanensis*	55.7–58.9	Lisdiyanti et al. (2000)	*Gluconobacter sphaericus*	59.5	Tanasupawat et al. (2011)
*Acetobacter oryzoeni*	53.1	Baek et al. (2020)	*Gluconobacter thailandicus*	55.3–56.3	Malimas et al. (2007)
*Acetobacter oryzifermentans*	52.4	Kim et al. (2018)	*Gluconobacter wancherniae*	56.6	Tanasupawat et al. (2011)
*Acetobacter pasteurianus*	51.8–54.3	Lisdiyanti et al. (2000)	*Gluconobacter uchimurae*	60.4–60.6	Tanasupawat et al. (2011)
*Acetobacter papayae*	60.5–60.7	Iino et al. (2012)	*Granulibacter bethensis*	59	Ramírez-Bahena et al. (2013)
*Acetobacter peroxydans*	59.7–60.7	Iino et al. (2012)	*Komagataeibacter europaeus*	56–58	Yamada et al. (1997)
*Acetobacter persicus*	58.7–58.9	Iino et al. (2012)	*Komagataeibacter hansenii*	58–59	Yamada et al. (1997)
*Acetobacter pomorum*	52.1	Sokollek et al. (1998)	*Komagataeibacter intermedius*	61.6	Yamada et al. (2000)
*Acetobacter senegalensis*	56	Ndoye et al. (2007)	*Komagataeibacter kakiaceti*	62.10	Škraban et al. (2018)
*Acetobacter sicerae*	58.3	Li et al. (2014)	*Komagataeibacter kombuchae*	59.63	Škraban et al. (2018)
*Acetobacter syzygii*	54.3–55.4	Iino et al. (2012)	*Komagataeibacter maltaceti*	62.5–63.3	Slapšak et al. (2013)
*Acetobacter tropicalis*	55.2–56.2	Lisdiyanti et al. (2000)	*Komagataeibacter medellinensis*	58–60.7	Castro et al. (2013)
*Acidomonas methanolica*	63–66	Ramírez-Bahena et al. (2013)	*Komagataeibacter nataicola*	62	Lisdiyanti et al. (2006)
*Ameyamaea chiangmaiensis*	66–66.1	Yukphan et al. (2009)	*Komagataeibacter oboediens*	59.9	Yamada et al. (2000)
***Asaia astilbes***	58.8–59.4	Suzuki et al. (2010)	*Komagataeibacter pomaceti*	62.53–62.75	Škraban et al. (2018)
*Asaia bogorensis*	59.3–61.0	Yamada et al. (2000)	*Komagataeibacter rhaeticus*	63.4	Dellaglio et al. (2005)
*Asaia krungthepensis*	60.2–60.5	Yukphan et al. (2004)	*Komagataeibacter swingsii*	61.7	Dellaglio et al. (2005)
*Asaia lannaensis*	60.8–60.9	Malimas et al. (2008)	*Komagataeibacter sucrofermentans*	62.33	Škraban et al. (2018)
*Asaia platycodi*	60.0–60.1	Suzuki et al. (2010)	*Komagataeibacter saccharivorans*	61	Lisdiyanti et al. (2006)
*Asaia prunellae*	58.9–59.3	Suzuki et al. (2010)	*Komagataeibacter xylinus*	59.4–63.2	Yamada et al. (1997)
*Asaia siamensis*	58.6–59.7	Katsura et al. (2001)	***Kozakia baliensis***	56.8–57.2	Ramírez-Bahena et al. (2013)
*Asaia spathodeae*	59.7–59.8	Kommanee et al. (2010)	***Neoasaia chiangmaiensisg***	63.1	Ramírez-Bahena et al. (2013)
***Bombella apis***	59.5	Yun et al. (2017)	***Neokomagataea tanensis***	51.2	Yukphan et al. (2011)
***Commensalibacter intestini***	36.85	Kim et al. (2012)	*Neokomagataea thailandica*	56.8	Yukphan et al. (2011)
*Commensalibacter papalotli*	36.66	Servin-Garciduenas et al. (2014)	***Nguyenibacter vanlangensis***	59.3–61.0	Vu et al. (2013)
***Endobacter medicaginis***	60.3	Ramírez-Bahena et al. (2013)	***Saccharibacter floricola***	51.9–52.3	Jojima et al. (2004)
***Gluconacetobacter aggeris***	65.4	Nishijima et al. (2013)	***Swaminathania salitolerans***	57.6–59.9	Ramírez-Bahena et al. (2013)
*Gluconacetobacter asukensis*	65.2–65.4	Nishijima et al. (2013)	***Swingsia samuiensis***	59.3–61.0	Malimas et al. (2013)
*Gluconacetobacter azotocaptans*	64.01–65.7	Nishijima et al. (2013)	***Tanticharoenia sakaeratensis***	64.5–65.6	Yukphan et al. 2008)

^a^The type species of each genus is indicated in bold

^b^Data taken from literature (for *Acetobacter*: Iino et al. 2012; Kim et al. 2018; Lisdiyanti et al. 2000, 2001; Malimas et al. 2007, 2008; Ramírez-Bahena et al. 2013; Suzuki et al. 2010; Tanasupawat et al. 2001, 2011; Yamada et al. 2000; Yukphan et al. 2004; for *Acidomonas*: Yamada et al. 1997; for *Ameyamaea*: Kommanee et al. 2010; for *Asaia*: Kim et al. 2012; Servin-Garciduenas et al. 2014; Škraban et al. 2018; Slapšak et al. 2013; Spitaels et al. 2013; Yun et al. 2017; for *Bombella*, *Commensalibacter*, *Endobacter*, *Gluconobacter*, *Gluconacetobacter*, *Granulibacter*, *Komagataeibacter*, *Kozakia*, *Nguyenibacter*, *Neoasaia*, *Neokomagataea*, *Saccharibacter*, *Swaminathania*, *Swingsia*, *Tanticharoenia*: from reference in table above)

#### *Acetobacter*

*Acetobacter* uses two membrane-bound enzymes (alcohol dehydrogenase (ADH) and acetaldehyde dehydrogenase (ALDH)) to oxidize ethanol to acetic acid during respiration, after which it further oxidizes acetic acid and lactic acid to carbon dioxide and water. However, *Acetobacter* are unable to utilize sugar alcohols such as glycerol, sorbitol, and mannitol to produce acetic acid. The respiratory chain coenzyme (CoQ) used by Acetobacter is Q9 (Kersters et al. 2006).

At present, the main strains used in industrial production of acetic acid in China are *A. pasteurianus* Zhongke AS1.41 and Huniang 1.01 (Chen et al. 2017), which are relatively uniform. Damage will occur in *Acetobacter* strains when the acetic acid concentration reaches 7–8%; therefore, these strains are mainly used in conventional surface production of vinegar and the final acid concentration usually does not exceed 8%, with a maximum acidity of 9–10% (Andrés-Barrao et al. 2016). A recent study reported that *A. pasteurianus* could produce acetic acid in a two-stage aeration protocol with a maximum acidity of 9.33% (Qi et al. 2014). In addition, strains isolated from traditional vinegars such as Chinese grain vinegar, Japanese Komesu and Kurosu vinegars, and South Korean black raspberry vinegar are mainly *A. pasteurianus* (Nanda et al. 2001; Song et al. 2016; Wang 2016).

#### *Komagataeibacter*

*Komagataeibacter* can oxidize ethanol to acetic acid and oxidize acetic acid to carbon dioxide and water (Yamada et al. 2012). The respiratory chain CoQ used by *Komagataeibacter* is Q10 (Kersters et al. 2006). Members of this genus are characterized by an absence of flagella and inability to produce brown compounds. In addition, some strains can produce cellulose, show an inability to produce 2,5-diketo-D-gluconate, are able to produce dihydroxyacetone from glycerol, and can oxidize glucose, galactose, xylose, arabinoside, and ethanol to produce organic acids.

*Komagataeibacter* strains, which can resist 15–20% acetic acid, are mainly used to produce fruit vinegar and alcoholic vinegar in liquid-state deep fermentation in Europe (Andrés-Barrao et al. 2016). Several *Komagataeibacter* strains have been isolated during high acidity vinegar production, including *K. europaeus*, *K. intermedius*, *K. oboediens*, and *K. hansenii* (Xia et al. 2016; Sievers et al. 1992; Trček et al. 2000; Yamada et al. 2012).

### Analysis of metabolic pathways in AAB

**Fig. 1:**
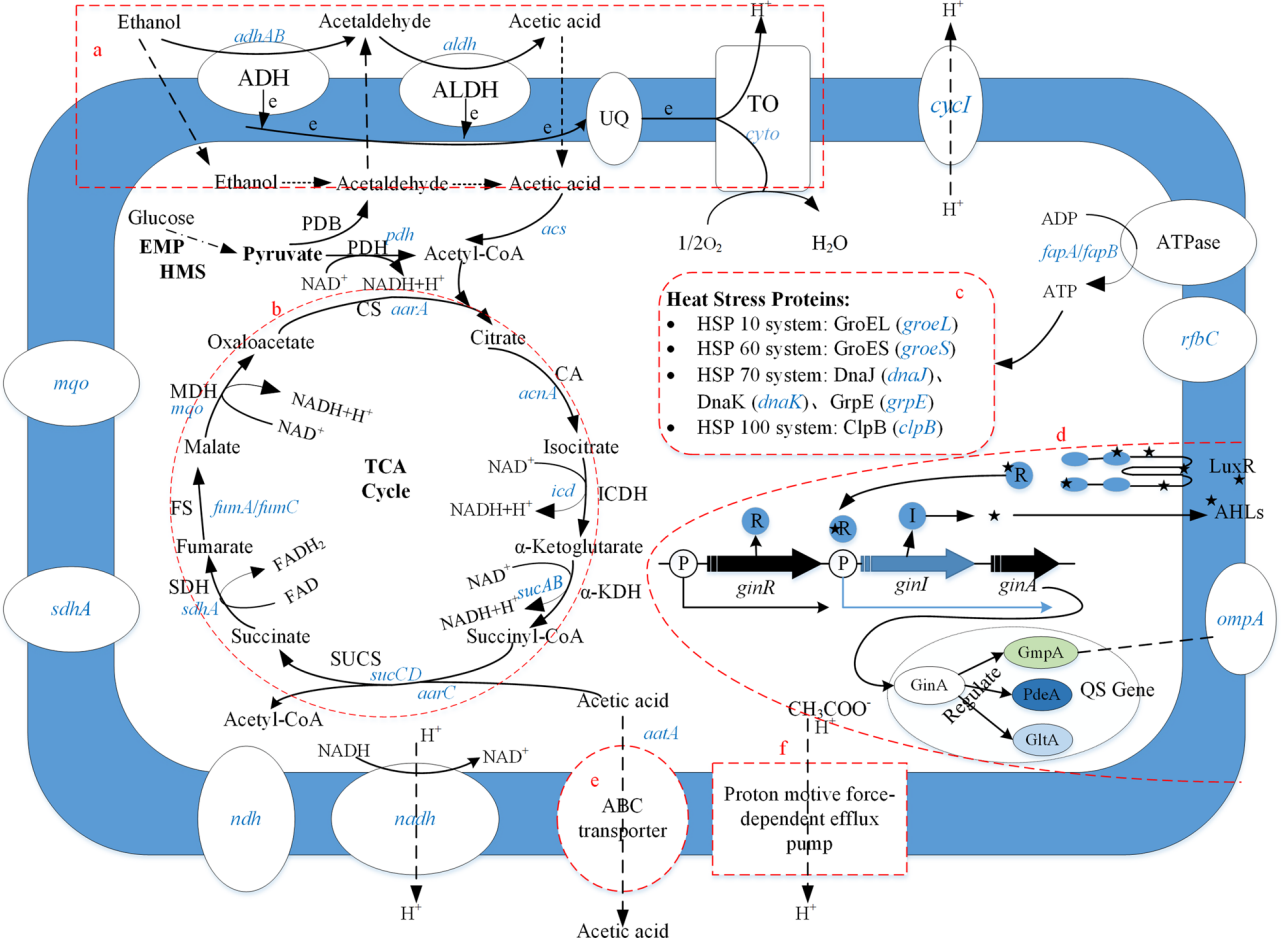
The metabolic pathways and mechanism of acetic acid resistance recorded in AAB. **a** The ethanol oxidation respiratory chain pathway; **b** The tricarboxylic acid (TCA) cycle pathway; **c** The heat stress proteins (HSPs); **d** A putative schematic representation of quorum sensing (QS) regulating modules in the cell membrane of *Gluconacetobacter intermedius*; **e** The ATP-binding cassette (ABC) transporter; **f** The proton motive force-driven efflux pumps. ADH, Alcohol dehydrogenase; ALDH, Acetaldehyde dehydrogenase; UQ, Ubiquinone; TO, Terminal oxidase; PDB, Pyruvate decarboxylase; PDH, Pyruvate dehydrogenase; CS, Citrate synthase; CA, Cis aconitase; ICDH, Isocitrate dehydrogenase; KDH, Ketoglutarate dehydrogenase; SUCS, Succinyl CoA synthase; SDH, Succinate dehydrogenase; FS, Fumarate synthase; MDH, Malate dehydrogenase; EMP, Glycolytic pathway; HMS, Hexose monophosphate shunt

Major metabolic pathways in AAB include the ethanol oxidation respiratory chain pathway, tricarboxylic acid cycle pathway, pyruvate metabolic pathway, and pentose phosphate pathway (Fig. 1). AAB possess unique oxidation capabilities, of which the classic reaction is incomplete oxidation of ethanol into acetic acid, which is used to produce vinegar.

This ethanol metabolic pathway consists of a two-step reaction (Mas et al. 2014; Tesfaye et al. 2002) (Fig. 1a). ADH present on the outer cell membrane in the respiratory chain facing the periplasmic space binds to pyrroloquinoline quinone (PQQ) to oxidize ethanol to acetaldehyde. Next, acetaldehyde is further oxidized by ALDH into acetic acid (Saichana et al. 2015). The entire process is an exothermic reaction, and oxidation of 1 mol of ethanol into acetic acid releases 493 kJ of heat (Matsutani et al. 2013).

In addition, AAB possess NAD-ADH and NADP-ALDH, which use NAD^+^/NADP^+^ as coenzymes. NAD-ADH and NADP-ALDH are located in the cytoplasm and can convert ethanol that enters the cell into acetic acid before converting acetic acid into acetyl-CoA to enter the tricarboxylic acid cycle for complete oxidation into carbon dioxide and water. PQQ-ADH and ALDH mainly participate in ethanol oxidative fermentation. During acetic acid synthesis, the activities of NAD-ADH and NADP-ALDH are completely inhibited (Chinnawirotpisan et al. 2003; Gullo et al. 2014; Yakushi and Matsushita 2010).

In addition to metabolic pathways for acetic acid synthesis, there are also many types of oxidation processes in AAB (Saichana et al. 2015). These reactions primarily occur on the cell membrane and are catalyzed by pyrroloquinoline quinone-bound dehydrogenase. In these reactions, substrates undergo incomplete oxidation to be converted into corresponding products that are released into the environment. Electrons released by different dehydrogenases are transferred to the terminal oxidase under the assistance of CoQ, which binds to oxygen (the final electron acceptor) to synthesize water. However, under hypoxic conditions other compounds can be used as the final electron acceptor to ensure bacterial growth (Drysdale and Fleet 1988). For example, acetaldehyde is used as the final acceptor in alcoholic vinegar fermentation, but microorganisms can only maintain physiological activity under this state and are unable to conduct acetic acid anabolism (Millet and Lonvaud-Funel 2000). Nevertheless, certain AAB are able to use quinones or vat dyes under hypoxic conditions, enabling respiration to continue (Qi et al. 2013).

Most metabolic pathways in AAB require oxygen, and oxygen consumption is directly proportional to acetic acid production. Hypoxia causes production capacities to rapidly decrease, and may even cause bacteria to die (Ory et al. 2002, 2004). Therefore, sufficient oxygen must be provided during fermentation.

### Acid resistance mechanisms in AAB

Acetic acid is a common weak acid that is used in biology and medicine. Acetic acid is highly toxic to microorganisms, with concentrations greater than 5 g/L inhibiting microbial growth and metabolism (Trček et al. 2015). The main reason acetic acid is toxic to microorganisms is its ability to cross the cell membrane and enter cells. This increases intracellular acetic acid concentrations and disrupts some physiological functions of the cell membrane (Conner and Kotrola 1995). There are large differences in acid resistance between different AAB species, with *K. europaeus* having high acid resistance and the ability to tolerate 15–20% acetic acid (Andrés-Barrao et al. 2016). In contrast, *A. aceti* and *A. pasteurianus*, which are commonly used in acetic acid fermentation, can only tolerate 5–8% acetic acid (Trček et al. 2007), while *Saccharibacter* and *Asaia* shows almost no growth in acetic acid-containing culture medium (Kommanee et al. 2011; Spitaels et al. 2013).

Acid resistance in AAB is intimately associated with cell structure and the levels of some enzymes in the cell membrane and cytoplasm. Acid resistance factors in *Acetobacter* and *Komagataeibacter* may be pyrroloquinoline quinone-dependent alcohol dehydrogenase (PQQ-ADH) and phospholipids on the cell membrane, proton motive force-dependent efflux pumps, ABC transporter, and some enzymes and stress proteins in the TCA cycle (Nakano and Fukaya 2008). In addition, some AAB are able to change their morphology and form pellicles on the cell surface to increase acid resistance. QS systems, which are present in *Komagataeibacter* and *Gluconacetobacter*, provide new ideas for acid resistance mechanisms in AAB from the signaling pathway perspective.

## Acid resistance factors on the cell membrane

### Cell morphology and cell membrane composition

Acetic acid has some effects on morphology in AAB. In the absence of acetic acid, *K. europaeus* appears as short rods. At an acetic acid concentration of 3% (v/v), *K. europaeus* appears as long rods and small depressions appears at the cell membrane. As acetic acid concentration increases, *K. europaeus* forms longer and thinner rods (Trček et al. 2007). Changes in cell morphology decreases the effective area for passive diffusion of acetic acid into cells and the toxicity of acetic acid accumulation in microorganisms, enabling them to tolerate high acetic acid concentrations.

*Acetobacter* and *Komagataeibacter* are acetic acid-producing strains commonly used in industrial processes, but the latter has higher acid resistance than the former. Analysis of the glycolipid and phospholipid content in *K. europaeus* revealed that the glycolipid content increased by 67% in bacteria growing in 3% (v/v) acetic acid culture medium compared with strains growing in culture medium without acetic acid while total phospholipid content decreased by 16.3% (Trček et al. 2007). Chemical analysis of cell membrane phospholipids revealed that the phosphatidylcholine (PC) content of *Komagataeibacter* was significantly higher than that of *Acetobacter*, while the phosphatidylethanolamine (PE) content was significantly lower than that of *Acetobacter* (Goto et al. 2000). As the acetic acid concentration increases, the proportion of PC in the cell membrane increases while the proportion of phosphatidylglycerol (PG) decreases (Higashide et al. 1996). By using the method of gene inactivation, it is proved that PC on the cell membrane is not only the main phospholipid component, but also an essential factor for high acid resistance in *A. aceti* (Hanada et al. 2001). A higher PC content and lower PE content are considered to be more favorable to the production of high concentrations of acetic acid.

When compared with other microorganisms, the cell membrane lipids of *Komagataeibacter* also contain high levels of carotenoids, particularly tetrahydroxybacteriohopane (THBH) (Matsushita et al. 2016). THBH is a characteristic component in the cell membranes of *Zymomonas mobilis*, and THBH content increases during alcohol fermentation (Hermans et al. 1991). The THBH content in the membrane lipid of AAB up to 25% and THBH contributes to the stabilization of cell membrane at high ethanol concentrations (Ebisuya 2015). Overexpression of squalene-hopen cyclase, which participates in the synthesis of THBH precursors, increased the acetic acid resistance of AAB compared with the wildtype strain which confirms that THBH is related to the acetic acid resistance in AAB (Ebisuya 2015).

Furthermore, *Acetobacter* can be classified by cell morphology as R (when the cell surface is rough) or S (when the cell surface is smooth) (Deeraksa et al. 2005). Studies have shown that the R strain can produce more acetic acid and possess acetic acid further oxidation ability compared to the S strain. The acetic acid further oxidation capacity is considered to be an important presentation of acid resistance in AAB, showing that the *A. pasteurianus* R strain has higher acid resistance than the S strain. In addition, the intracellular acetic acid/acetate content in the S strain is 3–4 times higher than that of the R strain, showing that acetic acid molecules can easily enter the cell membrane of the *A. pasteurianus* S strain due to absence of the pellicle (Kanchanarach et al. 2010). When the polE gene that is used for cell surface polysaccharide synthesis to confirm is deleted, the R strain has better resistance towards ethanol and acetic acid (Brandt et al. 2017). This is because the pellicle on the cell surface prevents the entry of acetic acid into cells and enables high concentration acetic acid fermentation.

### Enzyme activity level of pyrroloquinoline quinone-dependent alcohol dehydrogenase

PQQ is an important coenzyme that is ubiquitous in Gram-negative bacteria, participates in electron transport and can increase resistance towards radiation, high acidity, high temperature, and other extreme environments in certain microorganisms (Rajpurohit et al. 2008). PQQ-ADH is the key enzyme responsible for synthesizing acetic acid from ethanol in AAB (Fig. 1a).

The PQQ-ADH activity in highly acidic *K. europaeus* cells is two times greater than that of *A. pasteurianus* (Rajpurohit et al. 2008). When the ADH gene is deleted in *A. pasteurianus*, acid resistance is lost (Chinnawirotpisan et al. 2003), indicating that ADH activity contributes directly to acid resistance (Trček et al. 2007, 2006; Xia et al. 2015). Analysis of the genome of *A. pasteurianus* Ab3 revealed that it contains many membrane-bound PQQ-ADH (Wang et al. 2015c). Integrated analysis of published AAB genomes demonstrated that there are significant species differences in the gene copy number of PQQ-ADH. *Komagataeibacter* contains the most genes encoding PQQ-ADH and ADH, and these genes are absent in some strains of *Gluconobacter* and *Gluconacetobacter*. In addition, PQQ-ADH differs among species. Specifically, *K. europaeus* 5P3 contains six copies, while this gene is absent from *K. hansenii* ATCC 23,769 and *K. medellinensis* NBRC 3288. The copy number of PQQ-ADH in *A. pasteurianus* is relatively stable. Therefore, differences in the number of PQQ-ADH genes may be crucial to differences in acid productivity and acid resistance in different AAB strains (Wang et al. 2015a).

### ATP-binding cassette transporter

ABC transporter, which is located on cell membranes, is responsible for the transport of intracellular and extracellular substances and is ubiquitous in animals, plants, and microorganisms (Lewis et al. 2012). Currently, eight subfamilies of the ABC transporter superfamily have been identified based on amino acid sequences; namely, A, B, C, D, E, F, G, and H. Among these, the ABCA1 transporter, which participates in lipid transport and plays an important role in anti-atherosclerosis and promotion of cholesterol efflux in macrophages, is the most well studied (Lv et al. 2013).

A putative ABC transporter that affects acid resistance in *A. aceti*, is present on the cell membranes of *A. aceti* and is induced by acetic acid (Nakano et al. 2006) (Fig. 1e). Analysis of a series of proteins produced in *A. aceti* cell membranes and acetic acid by two-dimensional electrophoresis identified a 60 ku protein that is sensitive to acetic acid, which was named AatA. Molecular biology analysis of AatA revealed that it has a length of 591 amino acids, contains an ABC sequence and an ABC marker signal sequence, and that it belongs to the type B of ABC transporter subfamily (Linton and Higgins 1998). Comparison of AatA and macrolide transporters that are used as antibiotic efflux pumps revealed that they possess a common structure, showing that AatA may have a similar function as the latter (Kanchanarach et al. 2010; Méndez and Salas 2001; Mullins et al. 2012).

Studies have shown that aatA-deletion mutants have decreased formic acid, acetic acid, propionic acid, and lactic acid resistance. Disruption or deletion of the region between the two ABC transporters was found to lead to decreased acid resistance (Olano et al. 1995). Additionally, acetic acid resistance was restored if the plasmid pABC101 containing the aatA gene was inserted into aatA deletion mutants and acid resistance in *E.coli* containing pABC101 increased (Olano et al. 1995). These findings confirmed that aatA is an ABC transporter that is associated with acid resistance in bacteria and may act as an efflux pump for acetic acid (Nakano et al. 2006).

Comparative genomic analysis demonstrated that *Komagataeibacter* species contains more genes encoding putative ABC transporter proteins than *Acetobacter* (*K. oboediens* 174Bp2 possess 93 genes while *A. pasteurianus* IFO 3283–32 possesses 21 genes) (Wang et al. 2015a). This correlation demonstrates that strains with a high number of putative ABC transporter genes have higher acid resistance than strains with low numbers of putative ABC transporter genes.

### Proton motive force-driven efflux pumps

Studies of the acid resistance mechanisms in *Acetobacter* identified *A. aceti* that can adapt to high concentrations of acetic acid. The concentration of cytoplasmic acetic acid in this bacteria is significantly lower than that of AAB that cannot adapt to high concentrations of acetic acid. Some researchers speculate that the cell membrane may contain an efflux pump that can pump acetic acid from the cytoplasm out of the cell membrane (Fig. 1f).

To verify whether acetic acid efflux pumps are present in the cell membranes of AAB, Matsushita et al. (2005) employed isotope labeling to study the transport of acetic acid/acetate in intact *A. aceti* IFO 3283 cells and found that they possess an acetic acid efflux pump that is dependent on the proton motive force. In bacterial cells, acetic acid and two electrons are produced from ethanol under the action of ADH and ALDH (Matsushita et al. 2004; Nakayama 1961). The synthesized acetic acid undergoes passive transport from the periplasmic space, past the cell membrane into the cytoplasm. The synthesized electrons are then transported to the oxidase coenzyme to generate a proton motive force. By using the proton motive force, efflux pumps can pump intracellular acetic acid out of cells and prevent acetic acid accumulation from affecting the growth and metabolism of AAB, enabling them to tolerate a highly acidic environment.

This efflux pump does not act on ethanol and is vastly different from the classical ABC transporter Pdr12, which is used to transport acetic acid anions in yeast cells. Hence, this acetic acid efflux pump is a H^+^ retrograde transporter and not an ABC transporter (Matsushita et al. 2005). The acetic acid pump pumps out protonated acetic acid to maintain a low acetic acid environment in AAB.

## Acid resistance factors in the cytoplasm

### Overexpression of certain enzymes in the tricarboxylic acid cycle

A study found that AAB can oxidize acetic acid into carbon dioxide and water when the ethanol substrate in culture medium is exhausted to promote secondary growth (Matsushita et al. 2016). In this process, which is known as acetic acid assimilation, acetyl-CoA synthetase (acs) catalyzes the conversion of acetate to acetyl-CoA and citrate synthase (aarA). Acetyl-CoA then enters the TCA cycle, enabling the removal of acetic acid through the TCA cycle (Ramírez-Bahena et al. 2013) (Fig. 1b). *A. aceti* decreases the harmful effects of acetic acid accumulation through cytoplasm acidification, showing that the cytoplasm may possess substances that can adapt to an acidic environment.

Proteomics analysis of *A. pasteurianus* (4% (W/V)) and *Komagataeibacter* spp. (> 10%(W/V)) under acid stimulation revealed various proteins that play important roles in stress response, the tricarboxylic acid cycle, cell membrane modification, and outer membrane protein and cell morphology changes (Andrés-Barrao et al. 2012). Among these proteins, overexpression of enzymes involved in the tricarboxylic acid cycle, such as citrate synthase, isocitrate dehydrogenase, dihydrolipoamide dehydrogenase, succinate dehydrogenase, succinyl-CoA and CoA transferase (Andrés-Barrao et al. 2016), further confirmed the role of the TCA cycle in acid resistance in AAB.

To analyze the specific acetic acid resistance factors in the cytoplasm of AAB, analysis of proteomes induced by acetic acid was performed to detect genes and enzymes related to acid resistance. The results revealed that three genes (aarA, aarB, and aarC) will affect acid resistance in AAB and deletion of all three genes causes acid resistance to disappear in *A. aceti* 1023 (Fukaya et al. 1990). CS activity was not found in aarA gene deletion mutants of *A. aceti*, but introduction of aarA-containing plasmids restored CS activity. These findings demonstrated that the aarA gene is citrate synthase, which is closely associated with acid resistance in *A. aceti* (Mullins et al. 2008). Deletion of the aarC gene in *A. aceti* decreases acetic acid resistance and utilization capacities, but these two functions are restored after introduction of the aarC gene. In the TCA cycle, aarC replaces succinyl-CoA synthetase and directly converts succinyl-CoA to acetyl-CoA. The appearance of the branch can decrease the cell’s metabolic need for free CoA and regulate the effects of the TCA cycle on cytoplasmic pH (Francois et al. 2006). It is speculated that the aarB gene encodes the TCA activator SixA (Mullins et al. 2008). When there is a need to decrease intracellular acetic acid concentrations, these three aar genes synergistically act together to form a complete cycle that is different from the conventional TCA cycle (Fukaya et al. 1993). Large amounts of a 100 ku protein were found in acetic acid-containing culture medium, and sequence analysis revealed that it may be aconitase. Aconitase-overexpressing *A. aceti* can produce high acetic acid concentrations and decrease the growth doubling time. Increased aconitase activity and acid resistance was also found to increase the acetic acid concentration by 25%, which was a significant improvement in the fermentation productivity of acetic acid (Nakano et al. 2004).

The above studies confirmed that increasing the activity of one or more enzymes in the TCA cycle such as citrate synthase and aconitase will lead to rapid consumption of acetic acid or elimination of toxicity due to entry of acetic acid into the cytoplasm, causing intracellular acetic acid to be maintained at a low level and increasing acetic acid resistance.

### Heat stress proteins

Universal stress mechanisms are regulated by stress proteins known as molecular chaperones or chaperone proteins. HSPs are typical stress proteins that ensure correct folding of synthesized proteins in adverse environments and prevent intracellular protein denaturation (Hartl and Hayer-Hartl 2002).

GroES/L and DnaK/J are two common universal stress protein systems in AAB that are able to respond to many types of adverse environments (Yukphan et al. 2009). The HSP GroEL is significantly upregulated in *A. aceti* during batch feeding and continuous fermentation (Steiner and Sauer 2001). The transcript level of the groESL gene in *A. aceti* IFO 3283 was upregulated by heat, ethanol, and acetic acid. Furthermore, intracellular overexpression of the groESL gene can increase resistance to the aforementioned factors, showing that the groESL gene is related to resistance to adverse environments in AAB (Okamoto-Kainuma et al. 2002). Overexpression corresponding genes of intracellular grpE and dnaKJ increased resistance towards the fermentation environment in AAB (Ishikawa et al. 2010; Okamoto-Kainuma et al. 2004). Employing two-dimensional electrophoresis to conduct a comprehensive study of intracellular protein levels in *A. pasteurianus* LMG 1262 T during acetic acid fermentation, it was found that fermentation increased the protein expression levels of GrpE, DnaK, DnaJ, GroES, GroEL, and ClpB to varying extents, with the expression level of GrpE being increased by 9.42 times compared with the early fermentation stage (Andrés-Barrao et al. 2012; Wu et al. 2017). Overall, the aforementioned studies showed that the universal stress mechanism mediated by HSPs is one of the ways by which AAB ensure smooth acetic acid fermentation (Fig. 1c).

## Other factors

### Quorum sensing

Quorum sensing (QS) refers to the spontaneous production and release of specific signaling molecules by microorganisms in response to changes in the environment and sensing of changes in the concentration of these molecules for cell–cell exchange, thereby regulating the population behavior of microorganisms (Papenfort and Bassler 2016). Known major signaling molecules include: N-acyl-homoserine lactones (AHLs), autoinducer-2 (AI-2), diketopiperazines (DKPs), diffusible signal factors (DSFs), and 4-hydroxy-2-alkylquinolines (HAQs) (Mukherjee and Bassler 2019).

To date, QS in *Pseudomonas aeruginosa*, *Staphylococcus aureus*, *Pseudomonas fluorescens*, *Streptococcus mutans*, and *Helicobacter pylori* have been thoroughly investigated (Huang et al. 2009; Mukherjee et al. 2019; Rader et al. 2011; Wang et al. 2015b; Zhao et al. 2016), and studies have shown that QS plays important roles in biofilm formation, synthesis of virulence factors, and stress responses (Nickzad et al. 2015). However, research regarding QS in industrial microorganisms is relatively scant and has primarily focused on *Lactobacillus* (Maldonado-Barragán et al. 2009). The QS system has been found to be intimately associated with bacteriocin secretion and cathelicidin production during the growth of *Lactobacillus*. Additionally, QS plays an important role in cell morphology changes and changes in adhesion in response to adverse external environments (Kareb and Aïder 2019). Genome analysis studies have shown that certain AAB genomes possess homologous sequences that are similar to the luxI/luxR gene (luxI/luxR genes are usually homologues of ginI/ginR). Therefore, QS systems may play an important regulatory role in acid resistance, acid production, and growth of AAB.

The first AAB that was shown to possess a QS regulatory mechanism was *K. intermedius*, and Iida et al. (2008a) was the first to report the ginI/ginR QS system in *K. intermedius*. The ginI gene encodes an AHLs synthase and ginR encodes a signal sensor protein. Knocking out ginI or ginR genes can increase the growth rate of AAB in ethanol-containing culture media as well as increase acetic acid and gluconic acid productivity and defoaming ability (Iida et al. 2008a). Further analysis also showed that GmpA, a protein from the OmpA family, is regulated by QS systems and that this protein plays an important role in inhibition of oxidative fermentation of acetic acid. Moreover, GmpA is directly regulated by GinA, which is a protein with a length of 89 amino acids that is encoded by a gene located downstream of ginI (Iida et al. 2008b). This protein is unique to AAB and its expression is regulated by QS. In addition, the GinA protein can regulate the expression of other genes, including gltA, pdeA, pdeB, and nagA (Iida et al. 2009) (Fig. 1d). The culture medium of *Ga. diazotrophicus* PAL5 was detected eight QS signal molecules and confirmed that this organism possesses a QS regulatory mechanism (Nieto-Peñalver et al. 2012). In addition, whole genome sequencing showed that this bacteria possesses many signaling pathway encoding genes, including 16 c-di-CMP synthases, 14 membrane-bound histidine kinase signaling protein encoding genes, as well as a set of complete luxI/luxR QS encoding genes (Bertalan et al. 2009; Bertini et al. 2014). Subsequently, a study found that some genes in *K. xylinus* CGMCC 2955 jointly regulate intracellular c-di-GMP (a critical activator of the Bcs subunit) levels and confirmed the presence of the luxR gene (Liu et al. 2018). These findings demonstrate that QS is present in *K. xylinus* CGMCC 2955. Quenching of QS systems causes significant changes in the expression of intracellular and extracellular proteins, showing that QS systems may play an important role in population exchange, host colonization, and stress responses.

**Fig. 2:**
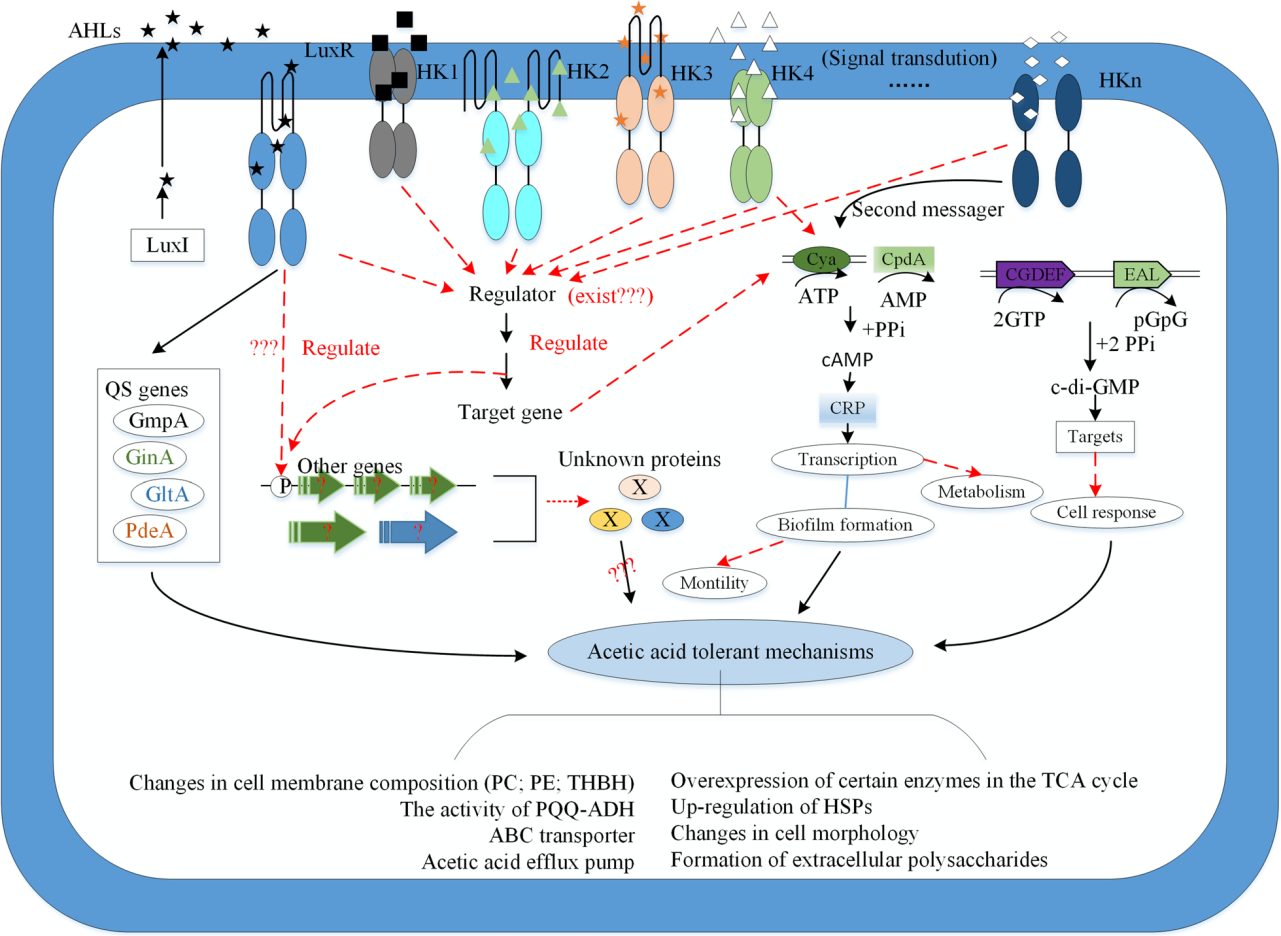
The possible quorum sensing system model in *Komagataeibacter* and *Gluconacetobacter*. The red dotted line and “?” refer to the potential relationship or the process have not been identified. The abbreviation of HK refer to histidine kinase

To date, QS regulatory mechanisms in AAB have only been found in *Komagataeibacte*r and *Gluconacetobacter* and there have been no reports of QS in other species. This is because the genome data of many AAB are incomplete. Additionally, even though sequences homologous to QS encoding genes are present in the genomes of AAB, the functions of these genes are mostly not annotated or they are hypothetical proteins. At the same time, the similarity of genes is low between different genera. Finally, AAB are highly variable, have unstable genomes, and different AAB may produce specific signaling molecules that cannot be detected based on existing detection methods. Although the potential correlation between QS system and other acid resistance mechanisms was proposed, there are still many processes that need more research to explain (Xia et al. 2017) (Fig. 2). In conclusion, QS research regarding *Komagataeibacter* and *Gluconacetobacter* has provided new data that will be useful for investigations of other AAB. With continuous improvements in AAB genomic data and annotation of new gene functions, signaling pathways regulated by acid resistance in AAB will be elucidated.

## Challenges and perspectives

Although AAB has long been used in vinegar fermentation, its incomplete oxidation characteristic has attracted increasing attraction owing to its potential in such applications as sugar alcohol oxidation, bio-fuel cells, and biosensors. Elucidation of acid resistance mechanisms in AAB is important to the selective breeding of AAB with high acid resistance and improving acetic acid fermentation processes. However, the acid resistance mechanisms in AAB are still not completely clear, as currently available data is insufficient for elucidation of the molecular mechanisms involved.

Too few proteins have been identified in proteomics to support global differential profile analysis, resulting in fragmentation and generalization of existing knowledge pertaining to acid resistance mechanisms. This poses challenges in construction of a complete pathway or process. In addition, further functional annotation of large amounts of unknown proteins is required. At the same time, the identification of low numbers of less abundant proteins, membrane proteins, and transcription factors also limits our understanding of how AAB respond to high acidity stress. Accordingly, further studies using more effective methods such as iTRAQ or MRM are needed.

QS systems provide new ideas for studying acid resistance mechanisms in AAB from a signaling pathway perspective. However, QS research regarding AAB is mainly focused on *Komagataeibacter* and *Gluconacetobacter*, and their intrinsic molecular regulatory mechanisms have not been fully studied. There is also an absence of studies confirming the distribution and regulatory pathways of QS in other AAB species. There are still many questions regarding the role of QS in regulating the physiological status of AAB and studies of genomics and metabolomics are needed (Fig. 2).

Other signaling pathways that are similar to the QS system, such as two-component systems and toxin-antitoxin systems, have been widely described in other bacteria and are known to be the major signaling regulatory networks. The regulation of acetic acid fermentation and acid resistance mechanisms in AAB by these other signaling pathways may be worth studying in the future.

## Data Availability

Not applicable.
